# Evaluation of prescription review and feedback policy on rational antibiotic use in primary healthcare settings in Beijing, China: a qualitative study using the Theoretical Domains Framework and the behaviour change wheel

**DOI:** 10.1093/jacamr/dlad128

**Published:** 2023-12-01

**Authors:** Haishaerjiang Wushouer, Kexin Du, Shicai Chen, Huangqianyu Li, Wanmeng Zhang, Yaoyao Yang, Lin Hu, Yue Zhou, Hui Sun, Bo Zheng, Xiaodong Guan, Luwen Shi

**Affiliations:** Department of Pharmacy Administration and Clinical Pharmacy, School of Pharmaceutical Sciences, Peking University, 38 Xueyuan Road, Haidian District, Beijing 100191, China; International Research Center for Medicinal Administration, Peking University, Beijing 100191, China; Department of Pharmacy Administration and Clinical Pharmacy, School of Pharmaceutical Sciences, Peking University, 38 Xueyuan Road, Haidian District, Beijing 100191, China; Department of Clinical Pharmacology, National Institute on Drug Dependence, Peking University, Beijing 100191, China; International Research Center for Medicinal Administration, Peking University, Beijing 100191, China; Department of Pharmacy Administration and Clinical Pharmacy, School of Pharmaceutical Sciences, Peking University, 38 Xueyuan Road, Haidian District, Beijing 100191, China; Department of Pharmacy Administration and Clinical Pharmacy, School of Pharmaceutical Sciences, Peking University, 38 Xueyuan Road, Haidian District, Beijing 100191, China; Department of Pharmacy Administration and Clinical Pharmacy, School of Pharmaceutical Sciences, Peking University, 38 Xueyuan Road, Haidian District, Beijing 100191, China; Department of Pharmacy Administration and Clinical Pharmacy, School of Pharmaceutical Sciences, Peking University, 38 Xueyuan Road, Haidian District, Beijing 100191, China; Department of Pharmacy, Peking University People’s Hospital, Beijing 100044, China; United Nations Children’s Fund, China Office, Beijing 100600, China; Institute of Clinical Pharmacology, Peking University First Hospital, Beijing 100034, China; Department of Pharmacy Administration and Clinical Pharmacy, School of Pharmaceutical Sciences, Peking University, 38 Xueyuan Road, Haidian District, Beijing 100191, China; International Research Center for Medicinal Administration, Peking University, Beijing 100191, China; Department of Pharmacy Administration and Clinical Pharmacy, School of Pharmaceutical Sciences, Peking University, 38 Xueyuan Road, Haidian District, Beijing 100191, China; International Research Center for Medicinal Administration, Peking University, Beijing 100191, China

## Abstract

**Objectives:**

To decelerate antibiotic resistance driven by inappropriate antibiotic prescribing, a prescription review and feedback (PRF) policy is implemented in primary healthcare institutions (PHIs) in Beijing, China. However, evaluation of PRF implementation in PHIs is scarce. This study aims to systematically identify the barriers and facilitators of PRF policy implementation to provide evidence for antimicrobial stewardship.

**Methods:**

We conducted key informant interviews with 40 stakeholders engaged in the implementation of PRF in Beijing, including physicians, pharmacists and administrators. Interviews were audio recorded and transcribed verbatim. We coded the interview transcripts and mapped informant views to domains of the Theoretical Domains Framework. We then used a behaviour change wheel to suggest possible behavioural interventions.

**Results:**

Procedural knowledge (*Knowledge*) and skills (*Skill*) of PRF were possessed by stakeholders. They felt responsible to promote the appropriate use of antibiotics (*Social/professional role and identity*) and believed that PRF could help to change inappropriate provider behaviours (*Behavioural regulation)* in prescribing antibiotics (*Beliefs about consequences*) under increased intention on antibiotic use (*Stages of change*). Moreover, informants called for a more unified review standard to enhance PRF implementation (*Goals*). Frequently identified barriers to PRF included inadequate capacity (*Skill)*, using punishment mechanism (*Behaviour regulation*), reaching consistently lower antibiotic prescription rates (*Goals*), lack of resources (*Environmental context and resources*) and perceived pressure coming from patients (*Social influences*).

**Conclusions:**

Stakeholders believed that PRF implementation promoted the rational use of antibiotics at PHIs in Beijing. Still, PRF was hampered by inconsistencies in review process and resources needed for PRF implementation.

## Introduction

Antibiotics are the most frequently prescribed therapeutic agents worldwide.^1,2^ Inappropriate antibiotic prescription drives antimicrobial resistance (AMR).^3^ Therefore, many interventions were introduced to alter antibiotic prescribing behaviours, for example, the electronic practice advisory tool,^4^ educational campaigns,^5^ public reporting,^6^ shared decision-making strategies,^7^ delayed prescriptions^8^ and prescription review and feedback (PRF).^9–11^ PRF is a commonly used method to review the appropriateness of prescriptions. It has been used in hospitals and proved effective to promote rational antibiotic use in the USA, France, Japan, and in low- to middle-income countries (LMICs), including Nepal, India and Lebanon.^9,11–16^ However, little evidence is available regarding the effectiveness of PRF in primary healthcare settings in China.

**Figure 1.**
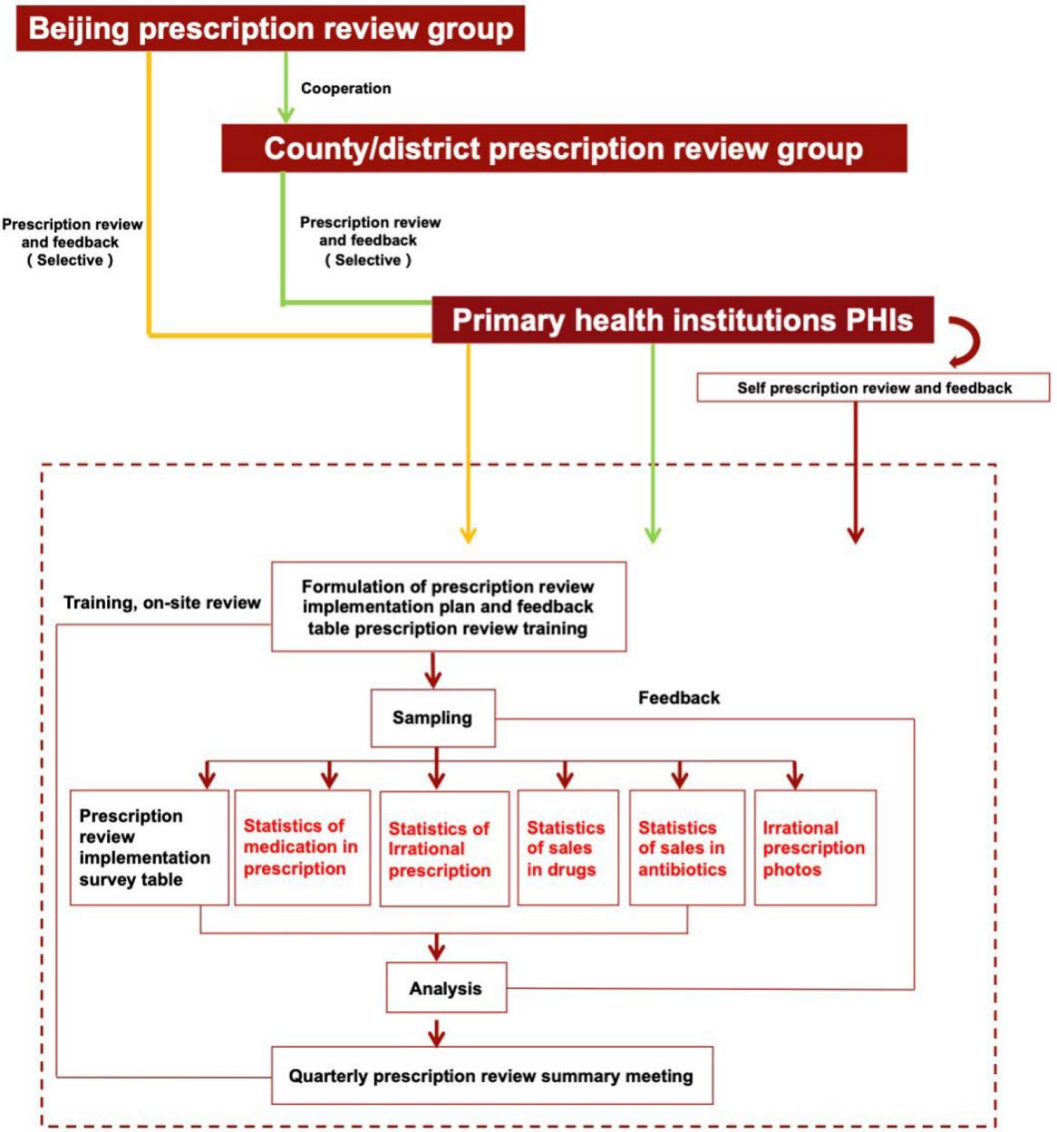
Mechanism of a three-level reviewing process of PRF policy implementation in Beijing.

In China, PRF was implemented as a pragmatic tool in primary healthcare institutions (PHIs) to constrain the inappropriate use of antibiotics, with Beijing being the first pilot area. A three-level PRF system (Figure 1) for PHIs has been established in Beijing since then. In 2017, the Beijing Municipal Health Commission (BMHC) issued the Action Plan on Promoting the Capacity of Primary Care,^17^ one of the core objectives of which was to strengthen the capacity of PHIs to conduct PRF. This included interventions of providing training programmes to promote the rational use of drugs based on national guidelines and incorporating PRF into performance appraisal management.

According to our previous study, after BMHC launched the PRF policy in 2017, decreases were seen in the antibiotic prescription rate (17.9% to 15.1%) and the irrational antibiotic prescription rate (14.0% to 9.2%) between 2017 and 2019.^18^ Moreover, a systematic review found that knowledge of factors associated with behaviour changes could address the barriers and enablers of antimicrobial stewardship (AMS), including audit and feedback in hospitals. In addition, social and contextual factors were also assessed as factors affecting antibiotic prescribing and engagement of AMS.^19^

As a core strategy of AMS, the implementation of PRF in PHIs is essential. Therefore, this study aims to systematically identify the barriers and facilitators of the implementation of PRF policy to provide evidence for further improvement.

## Materials and methods

This qualitative study was conducted according to the Standards for Reporting Qualitative Research (SRQR); the list content is shown in Table S1 (available as Supplementary data at *JAC-AMR* Online).

## Settings

As the capital of China, Beijing had 21.5 million residents and 2075 PHIs with 68.3 million visits in 2019 (25.8% of total hospital visits). PHIs are designed to be the first point of contact for patients with the national health system. All PHIs in Beijing are outpatient clinics with very little inpatient capacity (only 26 000 patients were discharged from PHIs in 2019), providing basic outpatient clinical care and public health services to individuals and families residing in the community.

To facilitate the implementation of PRF, the Beijing Prescription Reviewing System of Community Healthcare Institutions (BPRSCHI) database was established. The BPRSCHI database covered 327 PHIs. Sampling software is embedded in the information system of all PHIs. A total of 100 prescriptions were randomly selected from each PHI monthly using a systematic sampling method, with the sampling interval calculated by dividing the number of total prescriptions by 100. Due to the vast number of prescriptions resulting from numerous visits to Beijing, this methodology is used to select representative prescriptions for prescription review across the city.

## Recruitment of key informant interview targets

We used a three-step sampling method (Figure S1) to select the PHIs and therefore recruited physicians, pharmacists and administrators inside the PHIs for key informant interviews (KIIs). Furthermore, the purposive sampling technique was used in the recruitment of administrators from the District/County-level Department of Health and the Beijing Prescription Review (BPR) working group for KIIs.

## Data collection

Each informant from all 40 stakeholders participated in a KII (May 2021 to June 2021), and the interview was held online due to the COVID-19 pandemic. One member of the research team would ask questions of the informant and another research member would record the interview. Each interview lasted 30–60 min. The interviews focused on exploring the implementation, barriers and potential solutions to address inappropriate antibiotic prescriptions at PHIs. The interview content is shown in Text S1.

## Data analysis

All interviews were audio-recorded and transcribed verbatim using iFlyrec software. All analyses of interview research codes were coded for both technical and management perspectives and thematic/category content analysis was also conducted by two researchers (K.D. and W.Z.). Analysis of qualitative data on implementation facilitators and barriers of PRF was conducted using a deductive coding approach and through an iterative process to the constructs of the Theoretical Domains Framework (TDF). Occurrences of capabilities (C), opportunities (O) and motivations (M) model of behaviour change (B) (COM-B) components relevant behaviour change techniques (BCTs) were coded and mapped directly onto the relevant TDF domains to illustrate how the findings could be used to inform intervention design. The ranking of the domains we identified through the TDF is shown in Table S2. Specific codes for barriers and facilitators based on the TDF of each study are presented in Tables S3 and S4.

## Theoretical frameworks

**Figure 2.**
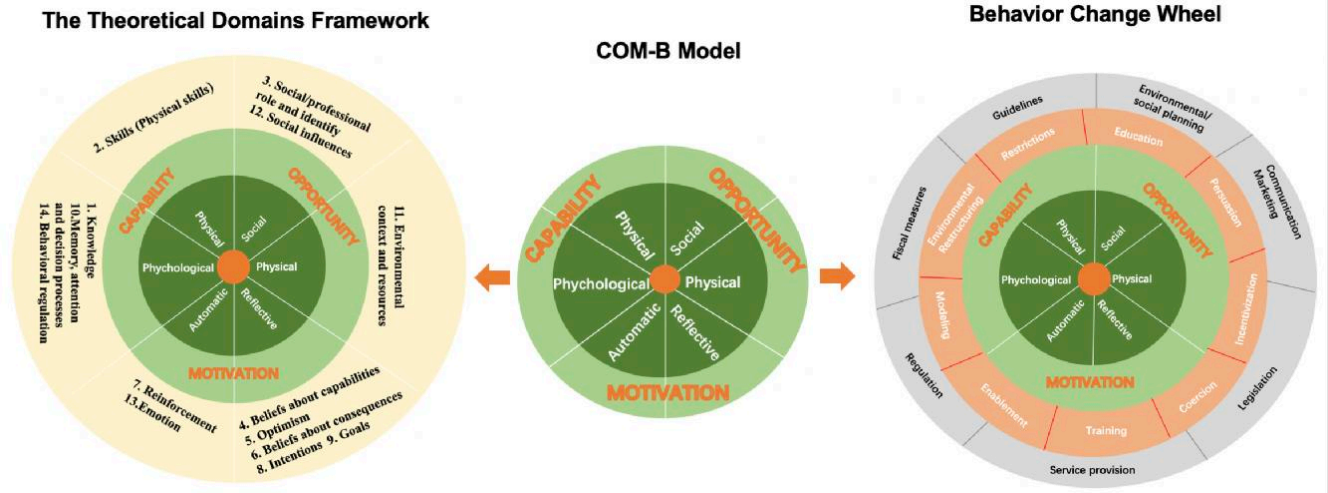
Domains of behaviour and behaviour change in Theoretical Domains Framework, COM-B and the behaviour change wheel.^20,21^

**Figure 3.**
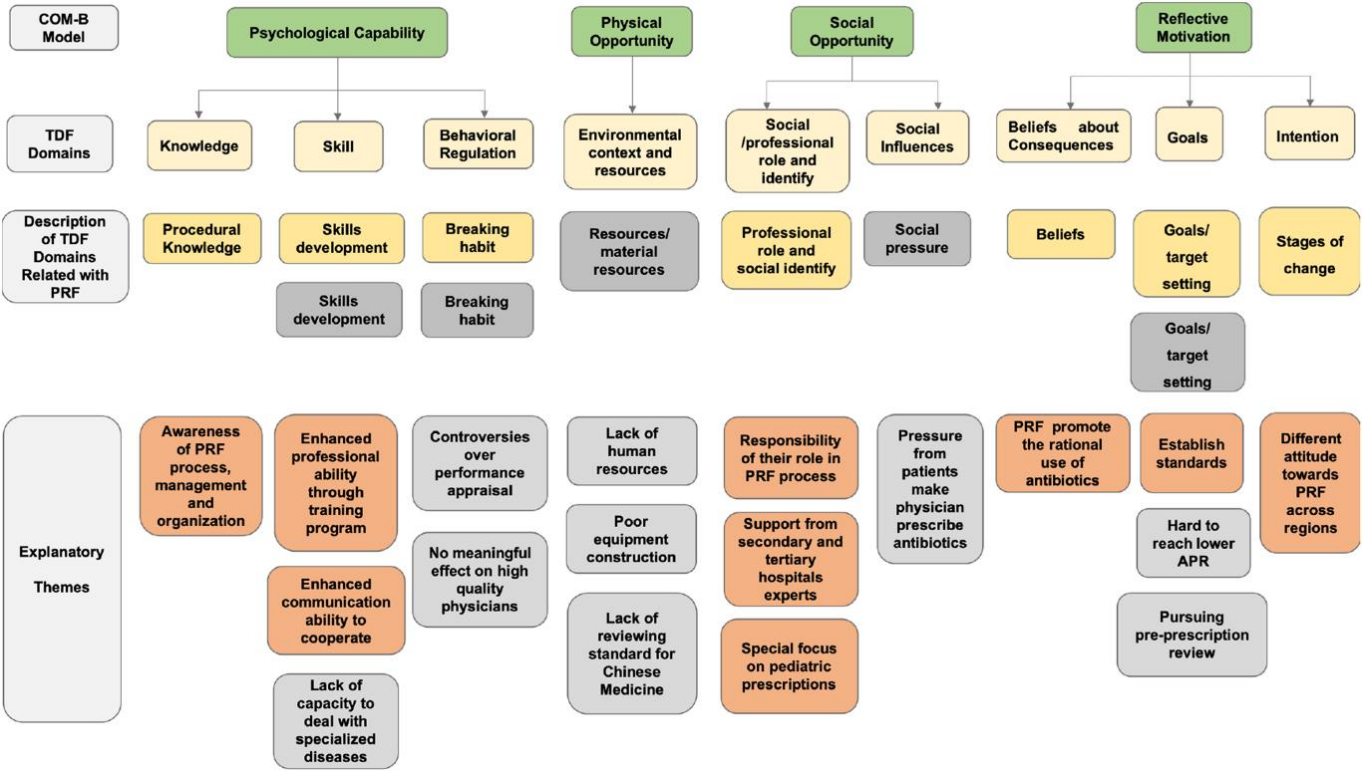
Barriers and facilitators of PRF implementation through TDF and the COM-B model.

The TDF is a framework derived from psychological theories to identify facilitators and barriers to changing physician behaviour, and has been used in a growing body of research.^20^ The TDF consists of 14 domains (Figure 2) that cover most of the psychological factors to explain the behaviour change of physicians during PRF intervention implementation. These domains expand upon the components of COM-B. The TDF and COM-B offer a systematic approach to identifying barriers and enablers of evidence-based practice, which allows for a theory-based development of interventions by selecting appropriate BCTs using a behaviour change wheel, intervention functions and policy categories that correspond to each domain^21^ (Figure 3).

## Ethics

Ethics committee approval was obtained from the Peking University Institution Review Board (IRB00001052-21048). Verbal informed consent with a waiver of documentation was approved by all participants to protect confidentiality.

## Results

### Characteristics of the qualitative interview data

We interviewed 40 stakeholders using semi-structured interview guides. Informants included 10 physicians, 14 pharmacists and 9 PHI administrators from these 12 selected PHIs for further interview. We also invited seven county-/district- and city-level administrators (Beijing Prescription Review Group) to participate in our study. The interview recordings were transcribed verbatim. Table 1 presents details of the informants.

**Table 1.** Number of interview targets

Title of interview targets	Frequency	Numbers
BPR working group administrator	1	N1
District/County administrator	6	N2–N7
Administrator of PHIs	9	N8–N16
Physician	10	N17–N26
Pharmacist	14	N27–N40

### The effect of PRF on antibiotic use

PRF policy was believed to promote the rational use of antibiotics since the antibiotic prescribing rate (APR) had been reducing for nearly 10 consecutive years in primary care. Systematic barriers and facilitators of PRF implementation are shown in Figure 3, and Tables S3 and S4.

### Facilitators of prescription review and feedback

#### Knowledge (procedural knowledge)

All stakeholders had heard that PRF was rolled out as a measure of AMS in their geographical area, while administrators were more knowledgeable about the management and organization of PRF in PHIs, like holding meetings to provide feedback on PRF work to pharmacists and physicians. Physicians and pharmacists were more knowledgeable about the actual procedures on how to review a certain prescription, such as applying drug labels, adhering to guidelines, and technical publications related to the infectious disease.

‘…conduct prescription reviews based on drug instructions, guidelines, and professional books related to prescription review’ (N32 Pharmacist)

#### Skills (skills development)

Both physicians and pharmacists recognized the importance of their professional ability to conduct PRF well. Therefore, administrators offered training sessions for pharmacists and physicians at PHIs to enhance their skills. For instance, physicians and pharmacists at PHIs could attend educational meetings with specialists from tertiary hospitals and online training programmes. Through the implementation of PRF, pharmacists and physicians were inspired to learn more about the rational use of antibiotics and to communicate with each other to better cooperate during PRF.

‘Through the PRF process, the physicians realized that they need to improve technical competence…’ (N27 District/County Administrator)

#### Social/professional role and identify (professional role and social identity)

All stakeholders acknowledged that PRF was critical to their work. Physicians and pharmacists generally felt motivated and responsible to promote the rational use of antibiotics through engaging in PRF. Pharmacists considered themselves ‘gatekeepers’ of care for patients.

‘…which can improve the clinical level of doctors, increase the knowledge base and better carry out our work.’ (N17 Physician)

Measures were taken at all levels to promote the appropriate use of antibiotics through recording and making public the unqualified prescriptions found in PRF. Furthermore, technical support was provided to PHIs from tertiary and secondary hospitals.

‘Review support was conducted by pharmacists from secondary and tertiary hospitals…’ (N5 District/County Administrator)

#### Beliefs about consequences (beliefs)

PRF conducted as a measure to promote inappropriate antibiotics was recognized by stakeholders, and PRF was considered an essential tool to enhance the quality of care as well as the pharmacy service at PHIs. Furthermore, administrators believed that PRF could help to intervene in the irrational clinical behaviour of inappropriate prescription of antibiotics.

‘Prescription reviews have a positive impact on antibiotic use, with antibiotic use declining every year for both adults and children.’ [N1 BPR working group administrator]

Positive consequences resulting from PRF implementation were mentioned by the BPR administrator, such as reducing the APR, reducing irrational antibiotic use, enhancing guideline adherence for diagnosis, enhancing the professional level of physicians and pharmacists in PHIs, and enhancing the trust between physicians and pharmacists, which would be favourable for the future transformation of pharmacists in PHIs in China from pharmacy services to clinical services.

‘Prescriptions are more standardized and rational, with fewer prescription errors and less duplication of medications.’ (N23 Physician)

‘Favourable to the future transformation of pharmacists is a good policy for pharmacy services.’ (N34 Pharmacist)

#### Intentions (stages of change)

The implementation of PRF led to an increased intention of antibiotic use among physicians, pharmacists and administrators to fulfil responsibilities pertaining to PRF to ensure rational antibiotic use, like using extra funds for PRF.

‘Some district health committees take it seriously and allocate specific funds for PRF.’ (N1 BPR working group administrator)

#### Goals (goal/target setting)

District administrators identified unified management processes were essential to establish a harmonized standard for conducting PRF. Moreover, one district-level administrator specifically pointed out the importance of designating specialized staff, ideally fulfilled by a clinical pharmacist or a paediatrician, as essential to conducting PRF successfully.

‘Beijing has unified a prescription pre-vetting platform to interface with the existing treatment systems of PHI to unify the review standards’ (N2 District/County Administrator)

#### Behavioural regulation (breaking habits)

Management tools targeted at the change of prescribing behaviour were developed to help physicians and pharmacists identify the inappropriateness of prescriptions. For example, performance appraisal was considered a major influence on physicians’ prescribing behaviours by all types of stakeholders alike. Under the pressure of performance appraisal, physicians would pay more attention to make sure prescriptions were appropriate to avoid being financially sanctioned.

‘Supervision of the uploading of this community health service is included in the performance appraisal to ensure the completion of the quality of this work.’ (N5 District/County Administrator)

To break irrational prescribing habits, facilities in the Haidian district had an initial review before conducting PRF to further identify and avoid medical errors during prescribing. For example, paediatric antibiotic prescriptions with a target APR ratio were used as a focus to correct irrational prescribing

‘Paediatric antibiotics are reviewed separately; a ratio for each department is developed…’ (N9 Administrator of PHI)

### Barriers to PRF

#### Skills (skills development)

The capacity of pharmacists at PHIs to review physicians’ prescriptions for patients diagnosed with specialized diseases was questioned by the administrators. Meanwhile, the inadequate capacity of pharmacists to communicate with physicians to correct irrational antibiotic use was admitted by administrators of PHIs as the bottleneck to conducting PRF.

‘Pharmacists and review specialists do not have a high enough level of expertise, and personnel have a lack of professionalism and may not have as much experience with more specialized diseases.’ (N13 Administrator of PHI)

#### Social/professional role and identify (professional role and social identity)

Pharmacists have an essential role during the PRF process. However, insufficient funding and professional staff hindered the promotion of pharmacists to invest themselves in PRF.

‘The number of pharmacists is low and the workload is high.’ (N32 Pharmacist)

#### Environmental context and resources (resources/material resources)

The lack of human resources (specifically pharmacists) and information systems limited PRF from reaching its full potential. In most PHIs, pharmacists only had Associate’s and Bachelor’s degrees and little work experience at large public hospitals, where freshmen were trained in more sophisticated programmes. Therefore, pharmacists in PHIs were only trained to dispense drugs and could have limited capacity to review prescriptions through a clinical lens. In addition, due to limited funding, the information system was not installed in some PHIs and therefore the pharmacists could only manually check the prescriptions, which was inefficient.

‘Pharmacists are required to juggle dispensing and prescription review duties, which puts more manpower pressure.’ (N33 Pharmacist)

Different management standards between districts or counties made it even more difficult to evaluate performance consistently across 16 districts in Beijing.

‘Performance review criteria may not be consistent across districts.’ (N1 BPR working group administrator)

The lack of equipment, systematic training and human resources were obstacles to better conducting of the PRF, as mentioned previously. Furthermore, when Chinese herbal medicines were involved in the prescription, it was hard to conduct PRF since there were no specific review criteria for this category of medicine.

‘There are no better and finer rules when it comes to pointing out herbal medicines; there are no more detailed criteria for drug interactions.’ (N13 Administrator of PHI)

#### Social influences (social pressure)

Patients and their family members were widely recognized as social influences on the prescribing of physicians. Sometimes physicians felt pressured by patients and their families to prescribe antibiotics.

‘Doctors will compromise to meet the needs of their patients and to not cause complaints. The community is simply using the drug for patient satisfaction.’ (N1 BPR working group administrator)

#### Behavioural regulation (breaking habits)

Obstacles were found from both management and technical perspectives. First, PRF results would affect performance appraisal, which was related to the salary of physicians in PHIs. This caused some controversies since some physicians would, therefore, pay more attention to their prescribing behaviour. However, some physicians were resistant to PRF, worrying that their salaries could be affected.

‘Performance-based intervention can be a double-edged sword that may cause some people to become rebellious and resistant to the prescription review process.’ (N16 Administrator of PHI)

## Discussion

To the best of our knowledge, this is the first study in the primary care setting to identify the systematic facilitators and barriers of PRF implementation in LMICs. Previous studies merely focused on the effect of PRF to reduce the inappropriateness of antibiotic use in a hospital setting, including optimizing carbapenem use by promoting a de-escalation strategy in Japan,^13^ increasing justified antibiotic use, de-escalation and adherence to guidelines in Nepal,^14,15^ decreasing antimicrobial use in India,^9^ and reducing antibiotic utilization in Lebanon.^16^ However, little attention was paid to how PRF, as a core strategy of AMS, exerts influence on the appropriateness of antibiotic use in primary care.^22^

In line with studies abroad, we found that some of the informants (42%) mentioned that PRF policy made antibiotic use more reasonable with the reduction in APR in nearly 10 consecutive years in the primary care setting (APR data from seven informants). Facilitators, including more prudent prescribing behaviour and attitude towards antibiotics, deeper communication with patients, and enhancement in expertise under management, were achieved through PRF. However, barriers to the expertise capacity of pharmacists and physicians, specialized human resources in PHIs, and discordant prescription standards across different districts still block the further enhancement of PRF.

Knowledge and skill were commonly documented factors in the well-studied knowledge-attitude-practice (KAP) model to exert a positive effect on physicians’ clinical choices when facing presumed infections in PHIs.^23–25^ A systematic review conducted in France show that, apart from knowledge and skill, other factors such as patient satisfaction and pressure, time constraints, diagnostic uncertainty and externalized responsibility could be driving factors of antibiotic prescribing decisions.^26,27^ Consensus of studies showed that a ‘culture of expectation’ could explain why patient satisfaction makes physicians compromise with an antibiotic prescription. Furthermore, the perception of patient pressure could be exacerbated by economic concerns or patient satisfaction scores.^27^ Time constraints resulting from work overload could be another factor in inappropriate antibiotic prescribing.^27^ Since symptoms of bacterial and viral infections could present similarly, diagnostic uncertainty could lead to antibiotic prescribing as a measure for physicians to avoid potential risks when diagnostic tests were not available.^26^

An umbrella review across PHIs worldwide showed that social influence factors could be a predictor of antibiotic prescribing of physicians, such as pressure from patients resulting from patient economic condition or other demographic characteristics.^28^ Likewise, our qualitative analysis also identified patient pressure as an underlying factor contributing to inappropriate antibiotic use. In addition, under the co-effect of patient influence under the ‘Social influence’ TDF subdomain and knowledge under the ‘Knowledge’ TDF subdomain, physicians would compromise an antibiotic prescription to alleviate the pressure from the patients. When considering the condition of paediatric visits, parental pressure such as reassurance and advice regarding children’s illnesses, along with other parental factors like poor antibiotic knowledge and personal past experiences influencing decision-making between parents and healthcare professionals,^29^ could cause an inappropriate prescription for children.

Barriers identified include a shortage of human resources and low capacity of information technology for pre-prescription review (Environmental context and resources) in PHIs in China. This finding is similar to what has been identified in other LMICs like India; non-clinical factors in this domain were found to contribute to antibiotic abuse, such as financial incentives and time pressure affecting the decision processes of physicians.^30,31^ Furthermore, factors such as physician beliefs about patient demands could affect the clinical decision of physicians, and inappropriate antibiotic prescribing would therefore drive antibiotic abuse.^32^ Furthermore, clinical guidelines in PHIs were issued by authorities around the world to conduct the right antibiotic use for clinical decisions. However, unsatisfactory adherence to clinical guidelines was found not only found in PHIs in LMICs, but also in PHIs in high- to middle-income countries (HMICs) like England and Denmark.^33,34^ Regarding medical as well as non-medical considerations, physicians developed and rely on their experience and habits in decision-making on antibiotic prescribing.^34^ This phenomenon highlighted the need for effective targeted intervention. For example, as our stakeholders implemented their PHI, they held a morning meeting to read up and share the Chinese guidelines for common infections (N10 Administrator of PHI), which enhanced the level of awareness and practice of adherence to the guidelines in the long run. However, lack of access to relevant antibiotic prescribing guidelines could also be considered a barrier in some HMICs.^35^

Overall, factors at the individual, community, health system and societal levels in mainland China were found to contribute to inappropriate antibiotic use in communities.^36^ Nonetheless, successful practices targeted at reducing and optimizing antibiotic prescribing could be reached under health system organizations and resources in Beijing, China.

Our study had limitations. First, the sample size was small due to the limited sources, which could undermine the representativeness of the results; however, we included all economic levels of all districts in Beijing and all types of stakeholders engaged in PRF to moderate the effect. Second, we did not provide the data on antibiotic resistance to show the consequence of PRF on prudent antibiotic use. Further study could focus on exploring the effect of PRF on antibiotic resistance. Third, the demographic data of the informants were not provided due to the limit of data accessibility.

### Conclusions

Our study identified facilitators and barriers to PRF implementation through the TDF framework in PHIs, in Beijing, China. However, focus on barriers is needed, including further improvement in harmonizing unified standards, and information technology, strengthening the guideline of PRF.

## Supplementary Material

dlad128_Supplementary_Data

## Data Availability

All data we generated or analysed in this qualitative study are provided in the Supplementary data.

## Supplementary data

Figure S1, Text S1 and Tables S1 to S4 are available as Supplementary data at *JAC-AMR* Online.
