# Supplementary material for: Evaluation of prescription review and feedback policy on rational antibiotic use in primary healthcare settings in Beijing, China: a qualitative study using the Theoretical Domains Framework and the behaviour change wheel
Source: JAC Antimicrob Resist. 2023 Dec 1;5(6):dlad128. doi: 10.1093/jacamr/dlad128 (PMC10691747; doi:10.1093/jacamr/dlad128)
Supplement: dlad128_Supplementary_Data [file dlad128_supplementary_data.docx]

**Supplementary materials**

**Table S1.** Standards for Reporting Qualitative Research (SRQR)a

| No | Topic | Item |
| --- | --- | --- |
| S1 | Title | Evaluation of prescription review and feedback policy on rational antibiotic use in primary healthcare settings in Beijing, China: a qualitative study using the Theoretical Domains Framework |
| S2 | Abstract | **Background:** To ameliorate the rapid development of antibiotic resistance driven by inappropriate antibiotic prescribing, prescription review and feedback (PRF) policy is implemented in Beijing, China. However, evaluation of PRF implementation in the primary healthcare setting is scarce. **Methods:** We conducted Key informant interviews (KIIs) with stakeholders engaged in all processes of PRF in Beijing, including administrators from physicians, pharmacists, and administrators of primary healthcare institutions (PHIs) as well as the administrator of the Beijing Prescription Review Group and District/County administrators. Views about the implementation of PRF were coded, with identified barriers and facilitators mapped to domains of the Theoretical Domains Framework (TDF) to enable comparisons between different stakeholders. **Results:** We conducted 40 KIIs with stakeholders of PRF implementation, 200 times barriers and facilitators of PRF implementation were mapped to 10 key TDF domains: “Knowledge”, “Skills”, “Social/professional role and identity”, “Optimism”, “Beliefs about consequences”, “Intentions”, “Goals”, “Memory, Attention and decision processes”, “Environmental context and resources”, “Social influences”. Among them, “Memory, Attention and decision processes”, “Beliefs about consequences”, and “Knowledge” were most often referred to as facilitators. “Environmental context and resources”, “Social influences”, and “Optimism” domains were most frequently mentioned as barriers. From both management and technical perspectives, PRF promoted appropriate prescribing of antibiotics, with consistent reductions in the antibiotic prescription rate (APR) for nearly ten consecutive years. **Conclusion:** The implementation of PRF in primary healthcare settings in Beijing contributed to more rational antibiotic use. However, barriers in technical and management perspectives still exist, further improvement in harmonizing unified standards, improving the knowledge of physicians and pharmacists, and developing information technology should be focused on. |
|  | **Introduction** |  |
| S3 | Problem formulation | Prescription review and feedback (PRF) is widely used to promote appropriate antibiotic use in low- and middle-income countries (LMICs), is a major driver of antimicrobial resistance (AMR). This work is the first to give a comprehensive overview of the barriers and facilitators of implementing PRF policy to address irrational antibiotic use in primary healthcare institutions in LMICs. |
| S4 | Purpose or research question | This study aims to (i) evaluate the implementation of PRF in primary healthcare settings (PHIs) in Beijing, China, and (ii) systematically identify barriers and facilitators of PRF implementation. |
|  | **Methods** |  |
| S5 | Qualitative approach and research paradigm | Information about barriers and facilitators of implementing PRF collected through key informant interviews were analyzed via the Theoretical Domains Framework. |
| S6 | Researcher characteristics and reflexivity | The research team conducted the interviews with the key informants, which may influence how the key informants respond during the interviews. Additionally, the research has no roles in relation to the implementation and review of the prescription review and feedback policy. |
| S7 | Context | This study is conducted in Beijing. As the capital of China, Beijing had 21.5 million residents (12.6% of whom were aged under 19) and 2,075 PHIs (including 345 community health centers (CHCs) and 1730 community health stations (CHSs) with 68.3 million visits in 2019 (25.8% of total hospital visits). All PHIs in Beijing are outpatient clinics with very little inpatient capacity (only 26,000 patients were discharged from PHIs in 2019), providing basic outpatient clinical care and public health services to individuals and families residing in the community. |
| S8 | Sampling strategy | We used a three-step sampling method (Table 1) to select the PHIs and therefore recruited physicians, pharmacists, and administrators inside the PHIs for key informant interviews (KII). Furthermore, the purposive sampling technique was used in the recruitment of administrators from the District/County-level Department of Health and the Beijing Prescription Review working group for KIIs. |
| S9 | Ethical issues pertaining to human subjects | Ethics committee approval was obtained from the Peking University Institution Review Board (IRB00001052-21048). |
| S10 | Data collection methods | Physicians, pharmacists, and administrators participated in an online one-on-one key informant interview (May 2021 to June 2021). Each interview lasted 30-60 minutes and was conducted by two members of the study team. The interviews focused on exploring the implementation, barriers, and potential solutions to address inappropriate antibiotic prescriptions at PHIs. |
| S11 | Data collection instruments and technologies | Interview is guided by the designed interview contents (questions). |
| S12 | Units of study | We interviewed 40 stakeholders using semi-structured interview guides including 10 physicians, 14 pharmacists, and 9 PHI administrators from these 12 selected PHIs for further interview. We also invited 7 administrators to participate in our study. |
| S13 | Data processing | All interviews were audio-recorded and transcribed verbatim using the software ifyrec (<https://www.iflyrec.com/>). Information collected through KIIs was analyzed using thematic analysis techniques. The analysis involved familiarization with full transcripts, developing initial codes based on constructs and themes emerging from the research questions and transcripts, refining codes, and allocating to broad themes. All analyses of interviews research codes were coded for both technical and management perspectives and thematic/category content analysis was also conducted. |
| S14 | Data analysis | Analysis of qualitative data on implementation facilitators and barriers of PRF was conducted by two reviewers) using a deductive coding approach and through an iterative process to the constructs of the TDF framework. |
| S15 | Techniques to enhance trustworthiness | Each interview was coded by 2 researchers using 3 rounds of grounded theory: open coding, axial coding, and selective coding. |
|  | **Results/findings** |  |
| S16 | Synthesis and interpretation | We found that nearly half of the informants (17/40) considered PRF policy made antibiotic use more reasonable with the reduction in APR in nearly ten consecutive years (APR data from seven informants). The effectiveness of PRF as a method to reduce inappropriateness of antibiotic use is proved in hospital settings in both LMICs and HMICs. 18-19 Through the lens of the TDF, we analyzed the effect on “Knowledge”, “Skills”,“Social/professional role and identity ”, “Optimism”, “Beliefs about consequences”, “Intentions”, “Goals”, “Memory, Attention and decision processes”， “Environmental context and resources”，“Social influences” domains of relative stakeholder by PRF. Facilitators like more prudent prescribing behavior and attitude towards antibiotics, deeper communication with patients, and enhancement in expertise under management were achieved through PRF. However, barriers to the expertise capacity of pharmacists and physicians, specialized human resources in PCCs, and discordant prescription standards across different districts still block the further enhancement of PRF. Additionally, we identified the relationships across subdomains，which showed complex interact within subdomains in inner and outer characteristics. |
| S17 | Links to empirical data | Not applicable. |
|  | **Discussion** |  |
| S18 | Integration with prior work, implications, transferability, and contribution(s) to the field | Knowledge and attitude which belongs to the “Knowledge” TDF subdomain and the “Skill” TDF subdomain were commonly documented factors in the well-studied knowledge-attitude-practice model to lean positive effect on the physicians’ clinical choices when facing presumed infections in PHIs. ^1-2^ We also found that the right knowledge regarding antibiotic use for respiratory infections both from the patient side and medical workers' side (like the physician and pharmacists) were essential to the right clinical choice of antibiotics in PHIs, same with the finding of Grossman Z and Cho H-J’. ^1-2^ However, knowledge could either be a barrier. In China, moves had been made to issue the knowledge level of health workers like continuing education and training lessons like advanced studies offered to refresher physicians or trainee physicians in PHIs. Besides that, when antibiotics are considered to be effective in treating a parent or a patient, they would ask for antibiotics from the physicians in PHIs. Apart from that, some even self-medicated themselves with antibiotics before their visits to PHIs. Meanwhile, the professional knowledge of a physician could influence the diagnostic ability, thus exerting an effect on his clinical judgment of antibiotic prescribing.^3-4^ Thereafter, lack of knowledge about antibiotic use, resistance, and infections of both physicians and patients, could result in inappropriate antibiotic use in PHIs.  An umbrella review across PHIs worldwide showed that factors under the “Social influence” TDF subdomain could be a predictor of antibiotic prescribing, like patient/condition characteristics, and patient influence.^5-6^ Same with this study, our qualitative analysis agreed with patient pressure as an underlying factor contributing to inappropriate antibiotic use. In addition, under the co-effect of patient influence under the “Social influence” TDF subdomain and knowledge under the “Knowledge” TDF subdomain, physicians would comprise an antibiotic prescription to alleviate the pressure from the patients. When considering the condition of pediatric visits, parental pressure such as the reassurance and advice regarding children's illnesses, with other parental factors like poor antibiotic knowledge and personal past experiences influencing decision-making between parents and healthcare professionals,^6^ which could cause an inappropriate prescription for children.  Barriers were found under the “Environmental context and resources” TDF subdomains like shortage of human resources and low capacity of information technology for pre-prescription review in PHIs in China. Same in other LMICs like India, non-clinical factors in this domain were found to contribute the antibiotic abuse, such as the financial incentives, and time pressure affecting the decision processes of physicians. ^7-8^ Besides that, factors like physician beliefs about patient demands, or physician's ignorance could be explained by the “Memory, attention and decision processes” TDF subdomain. These factors resulted in the clinical decision of physicians would therefore drive antibiotic abuse. ^7^ Moreover, a study in China found that the belief of antibiotic resistance driven by antibiotic use of physicians could influence their decision process of antibiotic prescribing. ^9^ Furthermore, clinical guidelines in PHIs were issued by authorities around the world to conduct the right antibiotic use for clinical decisions. However, not only in PHIs in LMICs but not satisfactory adherence to clinical guidelines was found also in PHIs in HMICs like England and Denmark. ^10-11^ Regarding medical as well as non-medical considerations, physicians developed and rely on their experience and habits in decision making on antibiotic prescribing. ^12^ This phenomenon highlighted the need for effective targeted intervention. For example, as our stakeholders implemented their PHI, they held a morning meeting to read up and share the Chine guidelines for common infections (N10 Administrators of PCC), which enhance the level of awareness and practice of adherence to the guidelines in a long run. However, lack of access to relevant antibiotic prescribing guidelines could also be considered a barrier in some HMICs. ^12^  Overall, factors at the individual, community, health system, and societal levels in mainland China were found to contribute to inappropriate antibiotic use in communities. ^13^ Nonetheless, successful practices targeted at reducing and optimizing antibiotic prescribing could be reached under health system organization and resources in Beijing, China. We used this comprehensive framework, which could explain the behavior change based on psychological factors thoroughly because antibiotic prescribing is as much behavior as a scientific decision. Moreover, we included different types of health professional stakeholders during the implementation of PRF, which is persuasive and representative. |
| S19 | Limitations | First, we only chose PHIs from Beijing as the site due to the limiting sources, but we include all economical levels of all districts in Beijing to moderate the effect. Second, we did not provide the data on antibiotic resistance to show the consequence of PRF on prudence antibiotic use. Further study could focus on exploring the effect of PRF on antibiotic resistance. |
| S20 | Conflicts of interest | The authors declare no conflict of interest. The sponsors had no role in the design, execution, interpretation, or writing of the study. |
| S21 | Funding | This study was funded by the United Nations Children’s Fund and the National Natural Science Foundation of China (Grant No. 81973294). |

**Figure S1.** Primary Healthcare Institution Selection

**
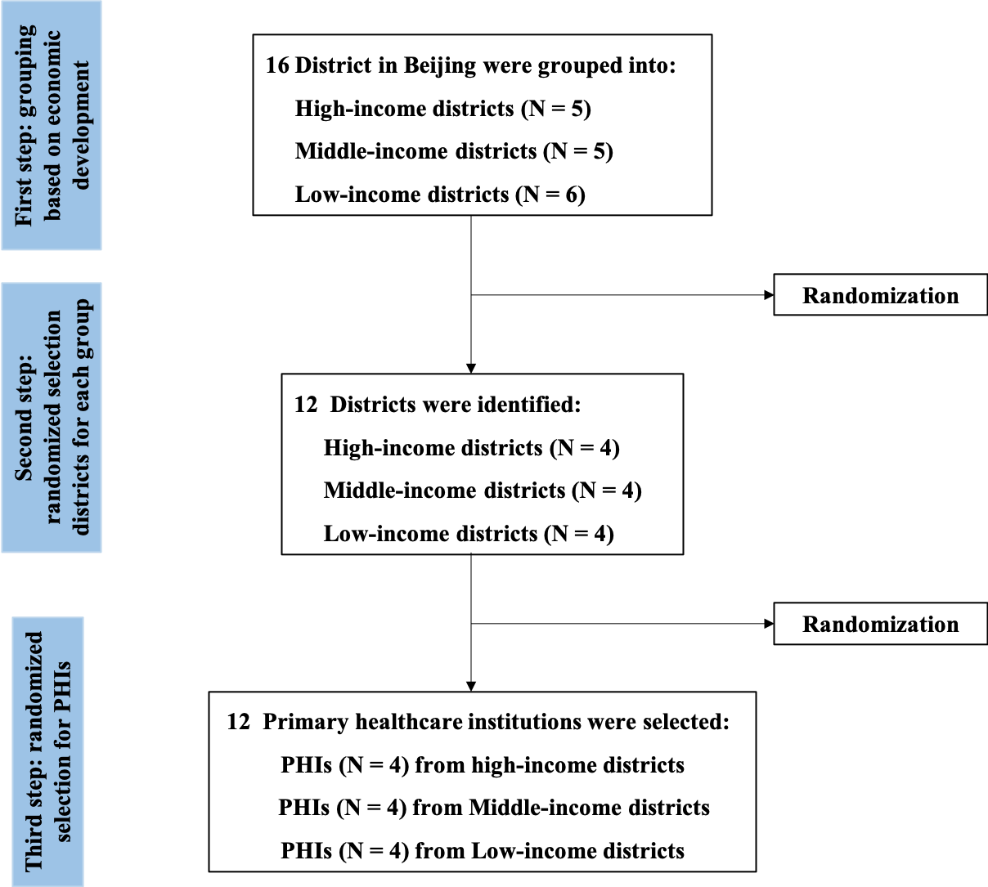
**

*Data was extracted from Beijing Statistic Yearbook 2019.

**Text S1.** Interview Content

*1. BPR working group administrators*

Warm-up: (1) The interviewers briefly introduce the background, purpose, information, and time of the interview, as well as the risks and benefits of the interview; (2) The interviewees signed the informed consent form; (3) The interviewees briefly introduced their positions/titles, work qualifications, main responsibilities, etc.

1.1 As a government leader, you are the policymaker of prescription comment. Please introduce the cooperation framework/coordination and cooperation mechanism among all levels of prescription comment work in Beijing, as well as the role of relevant personnel at all levels in the project.

1.2 Since the implementation of prescription comments for many years, have the relevant system and implementation rules been adjusted and improved? If so, what is it and why? What is the effect of adjustment or improvement?

1.3 Would you like to comment on the actual situation of prescription comment in various districts of Beijing, and how about the implementation effects of each district? Are there any differences in effects? If so, what might be the reason, and how do you plan to improve the areas where prescription comment doesn't work well?

1.4 Prescription comment involves doctors, pharmacists, and other workers in the medical system. What do you think of the performance of each part of the staff in the specific implementation process of prescription comment? Is there any need to adjust the work content of each part of the staff? If so, what are the reasons? How do you plan to manage and improve these problems?

1.5 What do you think of the effects of the support work of Beijing and district community comment working groups on the prescription comment of community medical institutions? If so, have the problems encountered by community medical institutions been solved in the process of support? If so, how is it solved?

1.6 Do you think there is a bottleneck in the current prescription comment in Beijing? If so, what is it and how do you suggest improving it?

1.7 Prescription comment has been implemented for many years, which has made a great contribution to the promotion of rational use of antibiotics in Beijing. What experiences and lessons do you think can be used for reference in the future?

1.8 Do you think the prescription comment in Beijing has improved the rational use of antibiotics in children in Beijing? If so, please talk about the impact of prescription comments on the rational use of antibiotics in children, and how prescription comments improved the rational use of antibiotics in children?

*2. District / County administrators*

Warm-up: (1) The interviewers briefly introduce the background, purpose, information, and time of the interview, as well as the risks and benefits of the interview; (2)The interviewees signed the informed consent form; (3)The interviewees briefly introduced their positions/titles, work qualifications, main responsibilities, etc.

2.1 As a district/County administrator, you are an important supervisor in the implementation of prescription comments. Please introduce the cooperation framework/coordination and cooperation mechanism among all levels of prescription comment work in your district/county, as well as the role of relevant personnel at all levels in the project.

2.2 Please comment on the overall implementation of prescription comment in your district/county, and how effective is it?

2.3 Are there any differences in the implementation effect of prescription comment among the community health service centers in your district/county? If so, what are the possible reasons and how do you plan to improve the underperforming areas?

2.4 Prescription comment involves doctors, pharmacists, and other medical system workers. What do you think of the performance of the staff in the actual process of prescription comment? How do you manage the relevant personnel?

2.5 Do you think there is a bottleneck in the prescription comment of your community service center? If so, what is it and how do you suggest improving it? Please talk about possible solutions.

2.6 Prescription comment has been implemented for many years, which has made a great contribution to the promotion of rational use of antibiotics in Beijing. What do you think are the characteristics/highlights of prescription comments in your district/county? What experiences and lessons can be learned in the future?

2.7 Do you think a prescription comment has an impact on the rational use of antibiotics in children? If so, please talk about how prescription comments affect the rational use of antibiotics in children.

*3. Administrator of PHIs*

Warm-up: (1) The interviewers briefly introduce the background, purpose, information, and time of the interview, as well as the risks and benefits of the interview; (2) The interviewees signed the informed consent form; (3) The interviewees briefly introduced their positions/titles, work qualifications, main responsibilities, etc.

3.1 As a manager of primary medical institutions, you are an important implementer of prescription comments. Please introduce the specific management structure, coordination, and cooperation mechanism of your organization, as well as the role of relevant personnel at all levels in the project. How do you manage people at all levels in prescription comments?

3.2 How do you ensure the implementation of prescription comments in your institution?

3.3 Please comment on the overall implementation and effect of prescription comment in your primary medical institutions.

3.4 The work of community prescription comment involves many medical system workers such as doctors and pharmacists. What do you think of the performance of the staff of your institution in the implementation process of prescription comment? Is there anything that needs to be improved? If so, how do you plan to improve?

3.5 In the process of implementation, has the municipal and district prescription comment working group provided technical support to your institution? If so, from which institutions and departments? Is there a feedback mechanism? If so, what is the specific situation? Does your district's prescription comment working group support and supervise your institution's prescription comment work? If so, how is it supported and supervised?

3.6 Pharmacists are the key to prescription comment. Do pharmacists in your institution have a unified comment standard before conducting comments? If so, what is the standard? How do you ensure that they have sufficient ability and relevant pharmaceutical knowledge to complete the comment?

3.7 Doctors are important decision-makers in prescribing. May I ask if the prescription comment of your institution has any impact on doctors? If so, please talk about it in detail.

3.8 Do you think there are bottlenecks in the implementation of prescription comments in your institution? If so, what is it and how do you suggest improving it?

3.9 Prescription comment has been implemented for many years, which has made a great contribution to the promotion of rational use of antibiotics in Beijing. What do you think are the characteristics/highlights of your institution's prescription comment? What experiences and lessons can be learned in the future?

3.10 Do you think a prescription comment has an impact on the rational use of antibiotics in children? If so, please talk about how prescription comments affect the rational use of antibiotics in children.

*4. Physicians*

Warm-up: (1) The interviewers briefly introduce the background, purpose, information, and time of the interview, as well as the risks and benefits of the interview; (2)The interviewees signed the informed consent form; (3)The interviewees briefly introduced their positions/titles, work qualifications, main responsibilities, etc.

4.1 As a doctor, you are the center of prescription comment. Do you know the prescription comment work of community medical institutions? Please talk about your understanding.

4.2 Does prescription comment affect you? If so, what is the impact? Please talk about it in detail.

4.3 Have you ever been informed that the prescription is unreasonable? Do you think the prescription comment result is correct? What would you do in this situation? If so, what are the main unreasonable prescriptions? How do you deal with unreasonable prescriptions?

4.4 Have you ever prescribed antibiotics for children? If so, what are the main factors you should consider before prescribing antibiotics? What antibiotics do you usually prescribe for children? Please talk about it in detail.

4.5 After the implementation of the prescription comment, has your prescription for children changed? If so, please talk about it in detail.

4.6 Do you think there is a bottleneck in the current prescription comment work of your institution? If so, what is it and how do you suggest improving it?

4.7 Do you think a prescription comment has an impact on the rational use of antibiotics in children? If so, please talk about how prescription comments affect the rational use of antibiotics in children.

*5. Pharmacists*

Warm-up: (1) The interviewers briefly introduce the background, organizational unit, purpose, methods, and requirements of the discussion; (2) The participants signed the informed consent form; (3) Participants of the forum will briefly introduce their positions/titles, working qualifications, main responsibilities, etc.

5.1 As a pharmacist, you are the center of prescription comments. Do you know the prescription comment work of community medical institutions? Please talk about your understanding.

5.2 If the work of commenting on prescriptions has an impact on you. If so, please talk about it in detail.

5.3 How do you comment on the prescription and whether you have participated in relevant training before? If so, what is the specific training? Are there any standards for implementation? If so, what are the standards for implementation?

5.4 When you comment on the prescription, have you encountered any unreasonable prescriptions? If so, what are the main unreasonable prescriptions? How do you communicate with doctors to improve their irrational prescription behaviors?

5.5 After the implementation of the prescription comment, have you found any changes in the situation of antibiotics prescribed by doctors for children? If so, please talk about it in detail.

5.6 Do you think there is a bottleneck in the current prescription comment work of your institution? If so, what is it and how do you suggest improving and solving it?

5.7 Do you think a prescription comment has an impact on the rational use of antibiotics in children? If so, please talk about how prescription comment affects the rational use of pediatric antibiotics.

## Table S2. Ranking of Theoretical Domain Framework (TDF) domains

| **TDF domain** | **Management view** | | **Technical view** | |
| --- | --- | --- | --- | --- |
|  | **Frequency (Facilitators)** | **Frequency (Barriers)** | **Frequency (Facilitators)** | **Frequency (Barriers)** |
| **Subtotal** | 61 | 24 | 102 | 54 |
| Knowledge | 15 | / | 11 | / |
| Skills | / | / | 16 | 6 |
| Social/professional role and identify | 12 | 19 | / | / |
| Optimism | 7 | 1 | / | / |
| Beliefs about consequences | 6 | / | 24 | / |
| Intentions | 3 | 1 | 5 | 1 |
| Goals | 9 | / | 9 | 1 |
| Environmental context and resources | / | / | 23 | 15 |
| Social influences | / | / | 5 | 26 |
| Behavior  regulation | 9 | 3 | 9 | 5 |

## Table S3. Facilitators and Barriers to Implementing an Antibiotic Prescription Review and Feedback Strategy in PHIs Based on the Theoretical Domains Framework from A Management Perspective

| **Domain** | **Sub-category** | **BPR working group administrators** | **District/County administrators** | **Administrators of PCCs** | **Physicians** | **Pharmacists** |
| --- | --- | --- | --- | --- | --- | --- |
| **Knowledge: an awareness of the existence of something** | | | | | | |
| Facilitators | Knowledge of management organization | Beijing Municipal Health Commission, Pharmacy and Pharmaceutical Department, and District Health Commission (N1) | Under the leadership of the Health Care Commission and the Medical Administration Department, and also under the work guidance of the Beijing Prescription Review Working Group, the work of prescription review and drug management is carried out (N2) | Under the direct management of tertiary hospitals, under the leadership of the pharmacy departments of tertiary hospitals, medical departments of community centers, and higher health authorities; under the collaborative guidance of community centers, with the establishment of community center pharmacy committees and quality control groups (N9) | - | - |
|  | Knowledge of the PRF content | The Pharmacy and Ordnance Division of the Beijing Municipal Health Care Commission is responsible for the publication of the notice, leading the performance assessment, and applying for research funding; the District Health Care Commission designates a unit (Social Management Center, Medical Administration Section, Pharmacy and Ordnance Section, and Medical Management Center) to help its community hospitals enter the review platform for online reviews (N1) | The Social Management Center provides specific supervision and supervises the community health service providers (N5) | Each month, members of the quality control team conduct regular inspections  Collaborative leadership of the pharmacy is equivalent to being under the relevant management of a tertiary care hospital (N8) | Apply expertise to develop work programs (N17) | Formulate center-specific review rules, medical insurance requirements, and policy requirements in compliance with relevant standards such as laws and regulations promulgated by the state; conduct prescription reviews based on drug instructions, guidelines, and professional books related to prescription reviews (N27, N31, N32) |
|  | Knowledge of PRF implementation timeline | Started in 2010, expanded the pilot by the end of 2014, and started to do the city in 2015, involving about 330 centers (N1) | The pilot began in 2010, and the prescription review was rolled out in 14 years throughout Beijing. In 2017, all 50 health service centers in Haidian District participated in Beijing's three-tier review. (N3) | - | Prescription reviews started around 2010 and have been a part of our community for ten years. (N19) | The earliest batch of prescription reviews in Beijing would have been in 2010. (N28, N39) |
| Barriers | - | - | - | - | - | - |
| **Skills: an ability or proficiency acquired through practice** | | | | | | |
| Facilitators | - | - | - | - | - | - |
| Barriers | - | - | - | - | - | - |
| **Social/professional role and identify:** **Professional identity/boundaries/role Group/social identity** | | | | | | |
| Facilitators | Conduct performance appraisal | Beijing Health Care Commission uses performance reviews as an assessment of rational drug use in hospitals in each district. (N1) | Review support was conducted by pharmacists from secondary and tertiary hospitals were conducted.(N5) | This prescription review is tied to our bonus, and failure to complete it will result in a performance penalty. (N9) | Physician prescribing behavior is associated with performance appraisal, which can improve the clinical level of doctors, increase the knowledge base and better carry out our work. (N17) | More emphasis is placed on prescription reviews, as they are linked to performance, and pharmacists are currently only linked to those in the prescription review team. (N33) |
|  | Linkage Management | - | - | Linkage of medical insurance, prescription, and clinical management to control prescription quality using medical insurance reimbursement (N11) | - | - |
|  | Establishing standards | - | - | We all have a system for developing our own center's management of this prescription review (N9) | - | The ratio of antibiotics used in each department is clearly defined (N36) |
|  | Realization of information technology | - | Interfaced with the city's community prescription review information system to automate information (N4) | We set up a workgroup and posted tasks inside the group. Every ten days, supervision is then conducted. (N12) | Change from manual entry to electronic entry. (N24) | The system draws 500 prescriptions per month to ensure a random sample. (N36) |
| Barriers | Insufficient funding support | Some districts and counties have less financial support (N1) | - | Lack of this dedicated funding to carry out this work. (N10) |  | Some pharmacists use their time off to work on reviews without pay. (N33, N36) |
|  | Inadequate staffing | Inadequate human resources. (N1) | There are differences in the degree of team building improvement and differences in the size of each community center. (N3) | The professional staff is mainly pharmacy staff, and there is a lack of clinical pharmacists. (N13) | Fewer pharmacists are invested in the staff. (N17) | The number of pharmacists is low and the workload is high. (N32) |
|  | Different management standards | Performance review criteria may not be consistent across districts (N1) | Shijingshan District to use the form of purchase of services, for the agency, it will be more important to the relationship of interest, management is more difficult. (N5) | - | Lack of a deeper management approach. (N19) | - |
|  | Lack of review criteria | - | - |  | The instruction dosage deviates from the standards of the prescription review. (N13,N18, N20) | There is no measure of drug interaction and no pathogenetic examination of the selected drugs. (N31) |
|  | There are obstacles to the promotion of information technology | - | Some companies' prescription pre-vetting systems are not compatible with the existing information systems of our community organizations. (N2) | - | Some primary care providers still conduct prescription reviews manually. (N19) | Some areas are not computerized due to economic conditions. (N37) |
| **Optimism: the confidence things happen for the best (including pessimism)** | | | | | | |
| Facilitators | Prescription reviews are useful | Prescription reviews are an important tool to promote quality of care. (N1) | This prescription review, is a prerequisite to promoting the quality of health care, to ensure the safety of the population's drug use. (N5) | I think the work of prescription reviews is very necessary. (N8) | I think the prescription review work is very meaningful, it not only regulates the behavior of our doctors but also, its main purpose is to promote the rational use of drugs. (N19) | (Prescription review) is a good policy for our entire pharmacy service. (N34) |
|  | Performance appraisal is useful | - | Prescription reviews have been incorporated into the performance appraisal, and errors in prescriptions are linked to performance, which is effective in this regard. (N3) | It has a positive effect because the control according to his performance is equivalent to disciplining him. (N16) | - | - |
| Barriers | Performance appraisal is not useful | - | - | Some people to become rebellious and resistant to the prescription review process since their pay will be affected. (N16) | - | - |
| **Beliefs about consequences: acceptance of the reality about outcomes of behavior in a given situation** | | | | | | |
| Facilitators | reduce antibiotic use | (Prescription reviews have a positive impact on antibiotic use), with antibiotic use declining every year for both adults and children. (N1) | The annual prescription reviews have been quite effective and have promoted rational drug use in the community. (N5) | Prescription Review is a great platform that intervenes not only in our medication use but also in our clinical behavior. (N11) | Antibiotics are being used less and less and are more symptomatic. (N22) | The proportion of antimicrobial use is more qualified, decreasing in quantity, decreasing in type, and decreasing significantly in use. (N28, N36) |
| Barriers | - | - | - | - | - | - |
| **Intentions: A conscious decision to perform a behavior or a resolve to act in a certain way** | | | | | | |
| Facilitators | Focus on prescription reviews | Some district health committees take it seriously and will allocate specific funds and conduct performance checks. (N1) | Both the leadership level and the level of the PHIs have taken this work more seriously. (N2) | The importance of prescription reviews was noted. (N14) | - | - |
| Barriers | Lack of attention to prescription reviews | Some districts and counties pay less attention, do not randomly sample, the number of prescriptions is small, and do not require the work of working groups and hospitals. (N1) | - | - | - | - |
| **Goals: mental representations of outcomes that an individual wants to achieve** | | | | | | |
| Facilitators | Management refinement | The Beijing government should refine the hierarchical management so that community hospitals have rules to follow. (N1) | - | Distinguish more finely between antibiotics, medications for the elderly, medications for children, medications for adults, and medications for patients with chronic diseases. (N11) | - | - |
|  | Unified Management | The performance of the city is uniform and the community hospitals are homogeneous. (N1) | Beijing has unified a prescription pre-vetting platform to interface with the existing treatment systems of community institutions. (N2) | - | - | - |
|  | Establishing standards | - | Establish guidelines to guide antibiotic prescribing or something like that. (N4) | Limit the number of outpatient antibiotics and promote antibiotic prescription reviews. (N13) | - | Establishing metrics for drug interactions. (N31) |
|  | Complete staffing recruitment | - | Each community health center tries to have a pediatrician as much as possible. (N3) | Establish dedicated posts to attract more talented pharmacists who can sink into the community. (N15) | - | - |
| Barriers | - | - | - | - | - | - |
| **Behaviour regulation：Anything aimed at managing or changing objectively observed or measured actions** | | | | | | |
| Facilitators | National policy | The decline in antibiotic use has also been influenced by national policies enacted. (N1) | - | Pediatric antibiotics are reviewed separately, a ratio for each department is developed in order to specifically focus on antibiotic use in children. (N9) | - | - |
|  | Performance Appraisal | Supervision of the uploading of this community health service is included in the performance appraisal to ensure the completion of the quality of this work；Conduct a performance appraisal system to regulate the behavior of doctors and pharmacists. (N1) | The good direct effect of performance appraisal (N6) | Prescription results are linked to physician bonuses. (N9, N12) | Linked to performance, it can regulate doctors' prescribing behavior to some extent. (N22) | Rewards and penalties for prescription reviews are linked to performance and are more effective. (N28, N32, N35) |
| Barriers | Unclear personal responsibility | - | We should emphasize team building and clear responsibility to facilitate accountability (N6) | Performance can be a double-edged sword that may cause some people to become rebellious and resistant to the prescription review process. (N16) | - | - |
|  | Lack of standards | - | The improvement of the effectiveness of the work carried out needs to be taken as a whole and lacks more extensive guidance (N4) | - | - | Different people have different criteria for reviews and there are subjective differences. (N27) |

## Table S4. Facilitators and Barriers to Implementing an Antibiotic Prescription Review and Feedback Strategy in PHIs Based on the Theoretical Domains Framework from A Technical Perspective

| **Domain** | **Sub-category** | **BPR working group administrators** | **District/County administrators** | **Administrators of PHIs** | **Physicians** | **Pharmacists** |
| --- | --- | --- | --- | --- | --- | --- |
| **Knowledge: an awareness of the existence of something** | | | | | | |
| Facilitators | Knowledge of the business implementation organization | Beijing Pharmacy Quality Control and Improvement Center for operational guidance (N1) | Beijing Prescription Review Working Group Guidance, Community Centers/Medical Institutions to Work (N2) | Establishment of Beijing Community Prescription Review Group, Pharmacy Committee, and Review Panel (N12) | - | - |
|  | Knowledge of the job content | The working group is responsible for work deployment, training, district rounds, supervision, recognition, performance evaluation, and establishing and maintaining a review platform. The community hospital is responsible for reviewing the platform, recording internal issues for feedback, internal training performance, and internal scheduling. (N1) | district review by a working group of pharmacists with high titles in community hospitals and secondary hospitals; and self-review within the institution/community center. (N3, N7) | The district health committee's social management center took the lead in setting up a district-level prescription review working group, which will regularly review the prescriptions uploaded by each community in the system, and also provide feedback on the results of the review in the system. A quarterly video meeting of the prescription review working group will be held to provide comprehensive and unified feedback on the district's prescription review work. (N12) | Prescription reviews are conducted at the end of the month. The system automatically reviews the prescriptions, the pharmacist reviews them twice, the expert committee reviews them again, the unqualified prescriptions are publicized and feedback is given, and a working group is established. (N20, N21,N24) | According to the relevant laws and regulations and technical specifications, we evaluate the standardization of prescription writing and the rationality of medication use, and if problems are found, we will intervene and develop improvement measures on time to promote the rational use of clinical medication. (N30) |
| Barriers | - | - | - | - | - | - |
| **Skills: an ability or proficiency acquired through practice** | | | | | | |
| Facilitators | High level of knowledge | - | Pharmacists and physicians need to have a certain knowledge base. (N2) | The knowledge of the pharmacist and the professional skills of the clinician is important. (N11) | There is a need for frequent study and frequent assessment, and we have to have competitions every year. (N19) | Through the PRF process, the physicians realized that they need to improve technical competence…(N27) |
|  | Strong communication skills | - | - | Physicians and pharmacists need to communicate fully. (N8, N13, N15) | Communicate with the pharmacy department, pharmacy, and the patient's family. (N17, N19, N22) | Pharmacists need to have healthy communication with physicians. (N28) |
|  | Cooperation | - | Both clinicians and pharmacists need to work well together and promote each other. (N3) | We have medical and pharmacy staff working very well with each other on a monthly quality control basis (N9) | If the pharmacist comes to point it out to you then surely you should cooperate to correct it in time. (N19) | Pharmacists and physicians need to work with each other. (N28) |
|  | Strong sense of responsibility | - | - | Hopefully, each of our pharmacists has a responsibility to not only dispense medication but also to know something very detailed and very cutting edge. (N11) | - | - |
| Barriers | Inadequate capacity | Some pharmacists and physicians are not competent. (N1) | There are differences in the titles, practice capabilities, and levels of doctors and specialists. (N6) | Pharmacists and review specialists do not have a high enough level of expertise, and personnel lack professionalism and may not have as much experience with more specialized diseases. (N13) | Physicians in PHIs are relatively less competent than those in large hospitals. (N20) | Pharmacists in PHIs may have a slightly lower level of competence. (N32, N38) |
| **Environmental context and resources：Resources/material resources or Environmental stressors or Person × environment interaction or Knowledge of task environment** | | | | | | |
| Facilitators | Conduct special reviews and full prescription reviews | Expanded prescription reviews for special reviews (antimicrobials) and specialty reviews. Conduct full prescription reviews on the files of contracted patients who use the most medications. (N1) | - | Take the entire prescription for this one person for a few months, a quarter, or even six months and read it against the medical record. (N11) | The prescription review team reviews monthly, with a specific review of children's prescriptions. (N21, N23) | - |
|  | Prescription pre-review | Community hospitals do prescription pre-review, and by extension, tertiary hospitals all do prescription pre-review. (N1) | The pre-review system that went online is running very smoothly so far and people are responding very well. (N2) | Using the prescription pre-review system, the pharmacy department will review the medication before dispensing it. (N8) | A pre-review system is used to review prescriptions before they are given. (N17, N26) | Use prescription pre-review to intercept some simple medication errors. (N35) |
|  | Training | - | Every year, the district has a training course for pharmacy staff, and some experts go down to the community to supervise and counsel. (N4) | Specialists in Beijing were invited to our center to train our physicians on some applications including traditional Chinese medicine and proprietary Chinese medicine. (N11) | The community management center and the Prescription Review Group held a special meeting to train us, and the Beijing Prescription Review Group also conducts related training several times a year. (N30) | One is to invite experts to do special training, for example, some knowledge of rational use of proprietary Chinese medicines. The other is for our pharmacists to promote some knowledge of rational drug use to doctors. (N28) |
|  | Feedback disclosure | - | - | We have created a hospital-wide physician group where the results of prescription reviews will be posted. (N12) Monthly performance will be publicized and we will all learn from each other. (N9) | Monthly summary of non-conforming prescriptions and public announcement. (N19, N26) | There is a daily summary of the prescription reviews, and the failed prescriptions will be publicized. (N31) |
|  | Well-staffed | - | Some communities are more fully staffed and have stronger teams built. (N3) | In terms of Western pharmacists and Chinese pharmacists, the composition ratio is still relatively reasonable. (N9) | - | - |
|  | Establishing a support group | - | The formation of a support group can help. (N4) | A morning study session was established to adhere to the prescription review process to ensure that they have sufficient competence or relevant knowledge to complete the review process. (N10) | - | - |
| Barriers | Lack of equipment | The economic level of each district varies, and some districts cannot support the installation of electronic sampling software resulting in regional differences in the uploading of prescription numbers. (N1) | Some districts are still using manual entry. (N5) | There are gaps in manual registration. (N15) | Some districts lack an informational approach and an automated screening system for prescription reasonableness. (N20) | The supporting laboratory tests cannot keep up with the rational choice of antibiotics has great constraints. (N27) |
|  | Insufficient human resources | Insufficient human resources (N1) | Variation in workload across community centers; insufficient number of pharmacists (N7) | There are human resource constraints (N14) | Less input from pharmacist staff in the community (N17) | Pharmacists are required to juggle dispensing and prescription review duties, which puts more manpower pressure. (N33) |
|  | Cumbersome refund process | - | - | The post-audit will involve the patient's return of the medication, and the patient may be unhappy, all of which creates certain difficulties in our work (N10) | - | Unreasonable prescription refunds are very inconvenient and the refund process is not smooth. (N28, N35, N36) |
|  | Inconsistent  reviewing  standards | Performance review criteria may not be consistent across districts (N1) | - | There are no better and finer rules when it comes to pointing out herbal medicines; there are no more detailed criteria for drug interactions. (N13) | - | - |
| **Behaviour regulation：Anything aimed at managing or changing objectively observed or measured actions** | | | | | | |
| Facilitators | Prescription reviews are useful | - | Raised awareness of rational drug use and improved prescription quality (N7) | I think the work of prescription review is very necessary (N8) | I feel that the prescription review process has had a positive impact (N23) | Prescription reviews are an important tool to improve clinical drug therapy (N30) and counteract the development of bacterial resistance. (N33) |
|  | Perceived positive impact on physicians, pharmacists | - | The doctor is supportive of the overall concept, and he is fully cooperative in his actions. (N15) | This has an impact on physicians and is one of the more positive ones. (N16) | It is still quite helpful to our work, which can improve the clinical level of doctors, increase the knowledge base and better carry out our work. (N17) | This has greatly promoted our pharmacists' motivation to learn and master new knowledge about prescription reviews and to do our duty as "gatekeepers" for patients in the community. (N31) |
| Barriers | Perceived ineffectiveness of prescription reviews | - | I think there is a lag in prescription reviews. (N5) | Post audit is not effective. (N10) | It is more rigid for some cases of over-indication of drugs. (N20) | - |
|  | Perceived little impact on physician prescribing | - | - | If the doctor is very self-disciplined, or the quality of medical care or pharmacist services is very high, perhaps the prescription review is not so meaningful to it. (N8) | I don't think the impact is too great. Our doctors are already more careful about prescribing pediatric antibiotics. (N22) | - |
| **Beliefs about consequences: acceptance of the reality about outcomes of behavior in a given situation** | | | | | | |
| Facilitators | Reduce antibiotic use | Antibiotic use is declining every year (for both adults and children) (N1) | The graded reviews and mutual support have reduced the use of antibiotics and increased awareness of rational drug use. (N3) | The prescribing rate for children has dropped significantly, and pediatric medication use has become more rigorous and rational. (N9, N13) | Antibiotics are being used less and less and are more symptomatic. (N18, N19) | Antimicrobial use has declined in number, decreased in type, and decreased significantly in use. (N28, N36) |
|  | Prescription failure rate reduced | Prescriptions are more reasonable. (N1) | The prescription failure rate was reduced and prescription quality was improved. (N2) | There has been a significant decline in our non-compliant prescriptions. Errors in routine dosage have largely disappeared. (N12) | Prescriptions are more standardized and rational, with fewer prescription errors and less duplication of medications. (N23, N24) | Improve prescription quality and reduce substandard prescriptions. (N32, N35) |
|  | Diagnostic specifications | - | - | Prescribing was more deliberate, and there was a marked improvement in physicians' routine errors. (N13, N14) | Comply with the rules of prescription reviews, prescribe strictly as required, and write prescriptions more standardized. (N19) | Antibiotics are not prescribed without bacteriological indications and are used according to the patient's changing condition. (N30) |
|  | Attitude specification | - | - | Physicians trust pharmacists more. (N8) | Consider more carefully, more cautiously, and more regularly, and do not use antibiotics lightly. (N17, N22) | Medication will be administered more carefully, medication guidelines will be strictly enforced, and unreasonable prescriptions will be communicated on time. (N31) |
|  | Facilitating the transformation of pharmacists | - | - | - | - | Favorable to the future transformation of pharmacists is a good policy for pharmacy services. (N34) |
| Barriers | - | - | - | - | - | - |
| **Intentions: A conscious decision to perform a behavior or a resolve to act in a certain way** | | | | | | |
| Facilitators | Intended prescription review | To further develop the prescription review process. (N1) | More attention is paid to medication guidance for patients. (N7) | Serious attitude, active participation, training, and communication. (N10) | Positive attitude. (N19) | High motivation. (N28) |
| Barriers | Not going to prescribe reviews | - | The expert reviews in some centers are not active, and there are differences in the degree of seriousness of the personnel. (N6) | - | - | - |
| **Goals: mental representations of outcomes that an individual wants to achieve** | | | | | | |
| Facilitators | Promoting Prescription pre-review | - | Implementation of prescription pre-review system, good system interface work, district level for funding (N5) | Hopefully, the pre-review of AI for pre-review, information systems intelligent review would work. (N9) | We hope to achieve automatic screening of prescription reasonableness and assist us in our daily prescription reviews. (N19) | Using artificial intelligence to install a prescription review system on the computer for doctors to achieve prescription safety risk indication and automatic review of rational drug use. (N39) |
|  | Making prescription reviews even better |  | - | Further, refine the process and carry out relevant auxiliary examinations. (N12, N14) | Further improvements can be made in the area of repeat medication in Chinese medicine. (N19, N22, N23) | - |
| Barriers | - | How to make the indicators carry out in-depth and continued to be more qualified is a question. (N1) | - | - | - | - |
| **Social influences: the interpersonal processes that can cause individuals to change their thoughts, feelings, or behaviors** | | | | | | |
| Facilitators | Leading by example | Experts go grassroots and help in depth. Exemplary hospitals take the lead. (N1) | Good work leads to poor results. (N4) | - | - | - |
|  | Good patient compliance | - | Patients are more compliant with their doctors than before. (N7) | - | Patient and explanatory work with patients. (N22) | - |
|  | National Policy | - | - | The 4+7 banded procurement and other national uniform procurement of drugs will affect this prescription of doctors. (N8) | - | - |
| Barriers | Patient Adherence | Doctors will compromise to meet the needs of their patients in order not to cause complaints. The community is simply going in to use the drug for patient satisfaction. (N1) | Patients may ask for medication somewhat by name (N4) | Our doctors tend to give in to parents' opinions because they have been involved in medical disputes or have medical conflicts. (N12) | Some parents may be a little resistant to testing and ask for antibiotics without testing. (N22) | Parents are anxious and will initiate a request for antibiotics. (N27) |
|  | Physician medication habits | - | Clinicians' prescribing habits and medication methods need to be improved, and physicians and pharmacists have different perspectives on their reviews. (N2, N5) | There may be problems with some of the habitual ways people use medication. (N13) | - | - |
|  | Pharmacist Level | - | There are differences in competence and levels between pharmacists. (N6) | Some practicing pharmacists will unconditionally obey their physicians and may just not adhere to their principles. (N11) | The knowledge level of rational use of proprietary Chinese medicines needs to be improved, and the review of Chinese medicine prescriptions is not detailed enough. (N17, N19) | Insufficient professional quality of primary pharmacists. (N38) |
|  | Inconsistent criteria for prescription review | Manual entry of prescriptions can be subjective and selective to make the results reasonable, resulting in poor quality prescription reviews (N1) | The rate of expert reviews is low and the perspective of expert reviews is not uniform enough. (N6) | There is no specific basis for the review of proprietary Chinese medicines, the indications are too broad, and it is difficult to control the repeated use of medicines. (N11) | The front-end review is completed by the drug instructions, and the prescription review is more rigid. (N17) | The limited amount of information in the prescriptions makes it difficult to judge the reasonableness of the medication and to understand the patient's medication history. (N27) |
|  | The low number of visits to children in the community | - | There are few pediatricians in the community and few pediatric patients, so relatively few antibiotics are used in pediatrics as well. (N3) | Some primary care hospitals do not have pediatric departments, and there are few prescriptions for children in outpatient clinics, and even fewer involving antibiotics. (N12) | - | Children still tend to go to larger hospitals, and there is a bias in our population of pediatric children. (N28) |
